# Anti‐Müllerian Hormone Concentrations Can Be Reliably Determined by a Single Measurement, Irrespective of Cycle, in Synchronised Ewes During Non‐Breeding Season

**DOI:** 10.1111/rda.70010

**Published:** 2025-02-21

**Authors:** Nebi Cetin, Davut Koca

**Affiliations:** ^1^ Department of Obstetrics and Gynecology, Faculty of Veterinary Medicine Van Yuzuncu Yil University Van Turkey

**Keywords:** AMH, anestrus, ewe, fertility, non‐breeding, reproduction

## Abstract

Improvement of yield characteristics in animal breeding is important in terms of increasing animal production and sustainability. Fertility is one of the most important yield traits affecting economic gain in sheep breeding. Anti‐Müllerian Hormone (AMH) is widely recognised as a dependable biomarker for assessing ovarian reserves and fertility potential. The aim of this study was to evaluate the dynamics of AMH during different phases of the sexual cycle in Norduz ewes the non‐breeding season. Additionally, the study sought to assess the effects of age and body condition score (BCS) on AMH concentrations during these phases. A total of 32 Norduz ewes with a body condition score (BCS) of 3–4.5 and aged between 2 and 4 years were used as animal material in the study. All experimental procedures were carried out outside the breeding season and when the ewes were lactating. In all ewes in anestrus, intravaginal sponges (Esponjavet, 60 mg MAP, Hipra, Turkey) were kept in the vagina for 7 days for estrus synchronisation. Intramuscular injections of PMSG (Oviser, 500 IU, Hipra, Turkey) and PGF2α analog (Gestavet, 50 μg, Hipra, Turkey) were administered 48 h prior to sponge removal. Twenty‐four hours after sponge removal, ewes were exposed to the ram for estrus detection. Since 5 ewes did not show estrus, blood samples were collected regularly from animals (*n* = 27) in which estrus was detected at three different stages: one just before the insertion of vaginal sponges (anestrus), another when heat was detected exposing to the ram (estrus), and the final one 10 days after estrus (diestrus). The serum samples were assessed for the levels of AMH and progesterone through the electrochemiluminessence immunoassay technique (ECLIA). The results of the analyses showed that serum AMH concentration did not vary between anestrus, estrus and diestrus phases of the sexual cycle of Norduz ewes outside the breeding season (*p* > 0.05). Furthermore, age and BCS had no effect on progesterone and AMH levels in different phases of the sexual cycle (*p* > 0.05). In conclusion, this study shows that serum AMH levels are constant at any stage of the estrus cycle. This suggests that phenotypic evaluation of ewes can be performed with a single measurement and that AMH is a reproducible and dependable biomarker that can be measured at any stage of the estrus cycle at an arbitrary time point.

## 1 Introduction

With the increase in the world population, the demand for animal production is increasing rapidly. This situation increases the interest in the development of new and innovative methods to meet the nutritional needs of the human population. However, reaching the desired levels of animal meat production can only be possible through correct and effective animal husbandry practices (Hamisi et al. 2023). In this context, the sheep industry has an important economic value in the livestock sector with meat, milk, wool and leather production worldwide (Reinoso‐Peláez et al. 2023). In addition, sheep play a critical role in sustainable agricultural practices with their ability to adapt to harsh environmental conditions and low maintenance costs (Armson et al. 2020). Fertility is a critical factor that directly affects profitability in sheep farming and is also one of the biggest challenges facing the sector (Reinoso‐Peláez et al. 2023). Therefore, careful selection of ewes with high fertility rates is of great importance to ensure the profitability and sustainability of sheep breeding (Molotsi et al. 2017; Nenova et al. 2023).

Anti‐Müllerian Hormone (AMH) belongs to the transforming growth factor β (TGF‐β) superfamily, which in mammals has a highly conserved dimeric glycoprotein structure of 140 kDa in total, composed of monomers weighing 70 kDa. (Cate et al. 1986; Pepinsky et al. 1988). It was first recognised as a hormone that regulates sexual differentiation in male mammalian foetuses by regression of Müller ducts (Jost 1947; Rey et al. 2003). AMH is produced by granulosa cells in pre‐antral and small antral follicles in the ovaries of females and controls folliculogenesis (Alward and Bohlen 2020; Monniaux et al. 2012). Since AMH is produced by healthy pre‐antral and early antral follicles, it is considered a dependable indicator of follicular reserve and has emerged as an important biomarker in reproductive biology (La Marca and Volpe 2006). Research in various species has provided important information on fertility by using AMH to assess antral follicle count (AFC) and ovarian reserve. This has been confirmed in many species, including women (van Rooij et al. 2002), cows (Ireland et al. 2011), goats (Alkan, Alkan and Kaymaz 2020) and sheep (Lahoz et al. 2014).

In female cattle, AFC is positively and highly correlated with peripheral blood AMH concentrations (Ireland et al. 2008). Similar to AFC, AMH concentration in plasma remains fairly constant in the same individuals throughout the estrus cycle and is not affected by seasonal changes. This feature facilitates the phenotyping of cattle with a single measurement in each season and at any stage of the estrus cycle (Ireland et al. 2008, 2011; Rico et al. 2009). These findings reveal that AMH concentrations can be dependably assessed with a single blood sample, independent of seasonal factors and days of the estrus cycle (Mossa and Ireland 2019). Thanks to this advantage, AMH has become a dependable biomarker for various purposes in cattle. These applications include determination of ovarian reserve and antral follicle populations (Ireland et al. 2008, 2011; Rico et al. 2009), evaluation of lifetime performance in the herd (Krassel et al. 2015), determination of age at puberty (El‐Sheikh Ali et al. 2017; Santa Cruz, Cushman and Viñoles 2018), determination of foetal sex (Stojsin‐Carter et al. 2017), determination of donor animals and evaluation of superovulation response (Jazmín et al. 2023; Mossa and Ireland 2019; Umer et al. 2019), estimation of fertility potential in young female cattle (Alward and Bohlen 2020) and identification of freemartin animals (Koca et al. 2024a).

Unlike cattle, the sexual cycle of small ruminants is seasonally related and they show anestrus. This process, called anovulation and ovarian quiescence, is different from the breeding season when ovulation and ovarian activity occur (Weems, Goodman and Lehman 2015). Due to these seasonal changes in the ovarian level, AMH levels in ewes may be expected to differ during anestrus compared to periods when the sexual cycle is active. However, this issue is still not fully elucidated and studies on the reproducibility of AMH during the off‐season and its use as a dependable biomarker are insufficient. Studies conducted during the breeding season in small ruminants have also confirmed that serum AMH levels can provide information such as determination of ovarian reserve and antral follicle populations (McGrice et al., 2020), selection of donor animals and evaluation of superovulation response (Alkan, Alkan and Kaymaz 2020; Lahoz et al. 2014), measurement of fertility potential (Turgut and Koca 2024a, 2024b). The use of AMH in sheep breeding, especially in reproductive management and genetic selection processes, has great potential. This feature will make it possible to evaluate individual reproductive performance in ewes and to make more accurate decisions in breeding selection. However, studies in sheep are not as comprehensive as in cattle and more research is needed.

Studies have been conducted on the relationship between AMH and various fertility parameters during the breeding season and AMH dynamics in the estrus cycle. However, AMH dynamics in ewes during the anestrus period, when sexual activity is absent, have not yet been sufficiently investigated. In this study, it was aimed to evaluate AMH dynamics by stimulating sexual activity in ewes outside the breeding season. In addition, it was aimed to examine the relationship between age and body condition score (BCS) and circulating AMH concentration in this period. With the data obtained, it is aimed to provide important contributions to the literature on whether AMH levels can be used as a dependable biomarker in ewes with only one blood draw, independent of seasonal factors and days of the estrus cycle.

## 2 Material and Methods

### 2.1 Ethics Statement

The study, consultancy and follow‐up of which was carried out by us, was conducted with the approved ethical guidelines of Van Yuzuncu Yil University (Protocol number 2024/04–19).

### 2.2 Location, Animals and Feeds

The study was conducted in a private small ruminant farm between April–May 2024. Location of the farm where the study was conducted is 38°35′42″ N 43°17′28″ E, Van, Turkey. All the experimental processes were performed outside the breeding season and when the ewes were lactating (suckling). A total of 32 Norduz ewes with a body condition score (BCS) of 3–4.5 and aged between 2 and 4 years were used as animal material in the study. The ewes were fed in accordance with the recommendations of the National Research Council (NRC). All animals were clinically healthy and free from reproductive issues. They were housed in a well‐ventilated shelter, ensuring adequate air circulation. Fresh water was provided ad libitum throughout the experiment. Feeding was carried out twice daily, in the morning and evening.

### 2.3 Estrous Synchronisation

**FIGURE 1:**
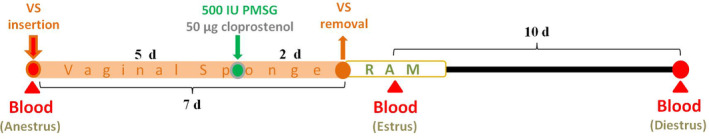
Estrus synchronisation protocol and blood sampling.

Estrous synchronisation in all ewes (*n* = 32) was performed using intravaginal sponges (Esponjavet, 60 mg MAP, Hipra, Turkey). The sponges were inserted for 7 days. Forty‐eight hours prior to sponge removal (on the 5th day of insertion), ewes received intramuscular injections of PMSG (Oviser, 500 IU, Hipra, Turkey) and a PGF2α analog (Gestavet, 50 μg, Hipra, Turkey) (Figure 1). For estrus detection, all ewes were exposed to a ram three times daily for at least 1 h each session over 3 days, beginning 24 h after sponge removal. Animals that allowed mating were identified and recorded, so that oestrus was detected in a total of 27 animals. Animals in which estrus was not detected (*n* = 5) were not used in the study.

### 2.4 Blood Sample Collection

Blood samples were obtained from the jugular vein of ewes at stages of the estrus cycle using with 5 mL capacity serum tubes. Particularly blood samples, one just before the insertion of vaginal sponges (anestrus), another when heat was detected exposing to the ram (estrus) and the final one 10 days following the estrus (diestrus) (Figure 1). Following collection, the blood samples underwent centrifugation at 3000 rpm for a duration of 15 min, and the serum was obtained and stored at −20°C for subsequent AMH and progesterone analysis.

### 2.5 Hormone Analysis

Serum samples were analysed to measure AMH and progesterone concentrations utilising the electrochemiluminessence immunoassay technique (ECLIA), as previously described (Marchetti et al. 2023; Koca et al. 2023; Turgut and Koca 2024a, 2024b). The analyses were performed using the Elecsys AMH and Elecsys progesterone automated assays on the Cobas 601 platform (Roche, Germany). Before the main study, method assessment was conducted according to the provided manufacturer instructions. Calibration and standard diagrams were carefully evaluated based on accurately allocated values. The assay showed analytical sensitivities of 10.0 pg/mL for AMH and 0.05 ng/mL for progesterone.

### 2.6 Statistical Analysis

All statistical analyses were conducted using Minitab (version 21.4.1) and Jamovi (version 2.3. 24). For the power analysis, the test power was calculated to be 80%, and the type‐1 error was fixed at 5% (G*Power statistics program, version 3.1.9.4). Anderson‐Darling normality test was used to evaluate distribution of the data. Given the normal distribution of the data, parametric tests were used. The impact of age and BCS on serum AMH and progesterone levels was assessed using one‐way ANOVA. Repeated measured analysis of variance (ANOVA) was used to evaluated differences in serum AMH and progesterone levels between different phases of the estrous cycle. The statistical significance level was defined as *p* < 0.05.

## 3 Results

Descriptive statistics of serum AMH and progesterone levels during cyclic phases are presented in Table 1. It was determined that serum AMH levels were between 80–440, 60–590 and 60–430 pg/mL and serum progesterone levels were between 0.05–0.35, 0.05–0.37 and 2.20–4.20 ng/mL during anestrus, estrus and diestrus, respectively (Table 1). Statistical analysis demonstrated that there was no significant difference in serum AMH levels between anestrus, estrus and diestrus phases of the sexual cycle (*p* > 0.05). However, serum progesterone levels were higher in the diestrus phase than in the anestrus and estrus phases, as expected due to luteal formation (*p* < 0.001).

**TABLE 1:** Descriptive statistics of serum AMH (pg/mL) and progesterone (ng/mL) levels according to sexual cycle phases.

Hormone	Phase	*n*	Mean ± SE	Minimum	Median	Maximum	*p*
AMH (pg/mL)	Anestrus	27	194.1 ± 17	80	170	440	> 0.05
	Estrus	27	205.6 ± 21.1	60	190	590	
	Diestrus	27	198.3 ± 18.1	60	180	430	
Progesterone (ng/mL)	Anestrus	27	0.17 ± 0.01^b^	0.05	0.19	0.35	0.000***
	Estrus	27	0.23 ± 0.01^b^	0.05	0.23	0.37	
	Diestrus	27	3.24 ± 0.11^a^	2.20	3.10	4.20	

*Note:* Different letters show significant differences between groups.

***p < 0.001.

The effect of age on serum AMH and progesterone levels during the cyclic phases is presented in Table 2 and the effect of BCS is presented in Table 3. Age and BCS had no effect on progesterone and AMH levels in different phases of the sexual cycle (*p* > 0.05).

**TABLE 2:** Effect of age on AMH (pg/mL) and progesterone (ng/mL) levels (Mean ± SE).

Age	*n*	AMH			Progesterone		
		Anestrus	Estrus	Diestrus	Anestrus	Estrus	Diestrus
2	15	176.67 ± 22.1	202.7 ± 32.2	190 ± 25.4	0.15 ± 0.02	0.21 ± 0.02	3.31 ± 0.16
3	6	193.33 ± 28.5	186.7 ± 33.2	193.33 ± 32.8	0.20 ± 0.04	0.25 ± 0.01	3.34 ± 0.29
4	6	238.33 ± 45.1	231.7 ± 42.6	224.17 ± 43.6	0.20 ± 0.01	0.25 ± 0.02	2.98 ± 0.16
	*p*	> 0.05	> 0.05	> 0.05	> 0.05	> 0.05	> 0.05

**TABLE 3:** Effect of BCS on AMH (pg/mL) and progesterone (ng/mL) levels (Mean ± SE).

BCS	*n*	AMH			Progesterone		
		Anestrus	Estrus	Diestrus	Anestrus	Estrus	Diestrus
3	7	158.57 ± 20.9	165.7 ± 27.5	156.4 ± 22.4	0.12 ± 0.02	0.18 ± 0.02	3.15 ± 0.26
3.5	11	223.64 ± 33.0	244.5 ± 42.6	235.45 ± 34.8	0.20 ± 0.02	0.25 ± 0.02	3.44 ± 0.15
4	4	200.0 ± 58.0	200.0 ± 55.8	205.0 ± 53.3	0.17 ± 0.06	0.23 ± 0.02	3.42 ± 0.19
4.5	5	174.0 ± 15.7	180.0 ± 24.5	170.0 ± 22.1	0.18 ± 0.01	0.23 ± 0.006	2.79 ± 0.31
	*p*	> 0.05	> 0.05	> 0.05	> 0.05	> 0.05	> 0.05

## 4 Discussion

AMH is recognised as an important biomarker for assessing ovarian reserve in animal species. In cattle, serum AMH levels showed marked changes during the prepubertal period, but remained relatively constant throughout the estrus cycle in the post‐pubertal period (Koca et al. 2023; Mossa et al. 2017; Umer et al. 2019; Yildiz et al. 2024). Also in sheep, AMH levels are not stable during the prepubertal period (Lahoz et al. 2012, 2014). However, studies conducted in the post‐pubertal period, during the breeding season, show that AMH levels are stable (Lahoz et al. 2014; Turgut and Koca 2024a, 2024b). Therefore, circulating AMH levels in the prepubertal period are not a suitable biomarker for assessing oocyte quality and ovarian reserve in ewes (Torres‐Rovira et al. 2014). It has been established through studies that AMH is strongly correlated with the number of healthy preantral and antral follicles. Moreover, cows with elevated serum AMH levels demonstrate a more pronounced superovulation response than those with lower AMH levels (Alward and Bohlen 2020; Koca et al. 2024b; McGrice et al. , 2020; Umer et al. 2019; Mossa and Ireland 2019).

It is reported that there is limited information in the literature on AMH levels during the estrus cycle in ewes (Waheeb 2017). However, it has been demonstrated that there is no change in serum AMH levels between the estrus and diestrus phases of the estrus cycle within the breeding season (Turgut and Koca 2024a, 2024b). The present study showed that there was no change in serum AMH levels in Norduz sheep during anestrus, estrus and diestrus phases in the non‐breeding season. This finding suggests that serum AMH levels are stable at any stage of the sexual cycle in accordance with previous studies in cattle and sheep. Furthermore, this proves that phenotypic evaluation of ewes can be performed with a single measurement and that AMH is a reproducible and dependable biomarker that can be measured at any stage of the estrus cycle, at an arbitrary time point.

In the study, serum AMH levels showed a wide variation among individual sheep (Table 1). In the literature, it has been reported that there may be differences in serum AMH levels between individuals within the same breed (Waheeb 2017; Turgut and Koca 2024a, 2024b). This variation is thought to be due to differences in ovarian reserves specific to each individual (Torres‐Rovira et al. 2014). In Norduz ewes, serum AMH levels varied between 80–440, 60–590 and 60–430 pg/mL during anestrus, estrus and diestrus periods, respectively. The mean serum AMH level was 205 pg/mL (Table 1). To the best of our knowledge, there is no available data in the literature on serum AMH levels of this breed. With this study, the serum AMH levels of Norduz sheep were added to the literature. In a previous study in Romanov sheep, it was reported that serum AMH levels ranged between 40 and 970 pg/mL and the mean AMH level was 329 pg/mL (Turgut and Koca 2024a). In another study conducted in crossbred Hamdani sheep, serum AMH levels varied between 95 and 520 pg/mL and the mean was 247 pg/mL (Turgut and Koca 2024b). These findings suggest that serum AMH levels may be affected by breed in ruminant species. When the data were evaluated, it was observed that Romanov sheep had higher serum AMH levels compared to crossbred Hamdani sheep. It was also reported that high serum AMH levels were associated with litter in Romanov ewes (Turgut and Koca 2024a), and associated with fertility in crossbreed Hamdani ewes were associated with reproductive performance (Turgut and Koca 2024b). In our study, it was found that serum AMH levels of Norduz sheep were different from the sheep breeds investigated in previous studies. These findings confirm that serum AMH levels may differ not only between individuals but also between sheep breed. Furthermore, these differences suggest that serum AMH levels may be related to ovarian reserve.

It was determined that age had no effect on serum AMH levels. And, AMH levels of 2‐4‐year‐old ewes did not change during the non‐breeding season in our study (Table 2). Similar findings were reported in previous studies conducted in sheep during the breeding season (Acharya et al. 2020; Turgut and Koca 2024b). Likewise, BCS (3–4.5) had no significant effect on serum AMH levels during different phases of the estrus cycle in Norduz ewes during the non‐breeding season (Table 3). Similarly, it was reported that BCS did not affect serum AMH levels in crossbred Hamdani ewes during the breeding season (Turgut and Koca 2024b). These findings suggest that both age (2–4 years) and BCS (3–4.5) are not factors affecting ovarian reserve in adult ewes outside the breeding season.

## 5 Conclusion

In this study, AMH levels were determined for the first time in Norduz breed sheep. Our findings indicates that serum AMH levels show large variations between Norduz ewes. In addition, it seems that breed is an important factor that may affect AMH level in sheep. In this study, age and BCS did not affect AMH levels. The findings of this study suggest that AMH levels can be dependably determined by a single measurement at a random time, regardless of the period of the sexual cycle in Norduz sheep. Overall findings of the study constitute an important step towards further fertility‐related studies investigating the use of AMH as a phenotypic biomarker in Norduz sheep.

## Author Contributions

Nebi Cetin: conceptualization; funding; investigation; methodology; resources; validation; visualisation; software; writing – original draft and writing – review and editing. Davut Koca: conceptualization; investigation; methodology; resources; writing – original draft and writing – review and editing and supervision.

## Conflicts of Interest

The authors declare no conflicts of interest.

## Data Availability

The data sets generated for this study are available on reasonable request from the corresponding author.
